# Reliability Study of c-Si PV Module Mounted on a Concrete Slab by Thermal Cycling Using Electroluminescence Scanning: Application in Future Solar Roadways

**DOI:** 10.3390/ma13020470

**Published:** 2020-01-19

**Authors:** Firoz Khan, Béchir Dridi Rezgui, Jae Hyun Kim

**Affiliations:** 1 Center of Research Excellence in Renewable Energy (CORERE), King Fahd University of Petroleum and Minerals (KFUPM), Dhahran 31261, Saudi Arabia; firoz.khan@kfupm.edu.sa; 2 Research and Technology Center of Energy, Photovoltaic Laboratory, Borj Cedria Science and Technology Park, BP 95 Hammam−Lif 2050, Campus Universitaire de Tunis El Manar, University of Tunis El Manar, Tunis 2092, Tunisia; bechir.rezgui@crten.rnrt.tn; 3 Division of Energy Technology, Daegu Gyeongbuk Institute of Science & Technology (DGIST), 333, Techno Jungang−Daero, Hyeonpung−Myeon, Dalseong−Gun, Daegu 42988, Korea

**Keywords:** renewable energy, thermal cycling, solar roadways, PV degradation, EL scanning, PV cell parameters

## Abstract

Several tests were conducted to ratify the reliability and durability of the solar photovoltaic (PV) devices before deployment in the real field (non-ideal conditions). In the real field, the temperature of the PV modules was varied during the day and night. Nowadays, people have been bearing in mind the deployment of PV modules on concrete roads to make use of the space accessible on roads. In this regard, a comparative study on the failure and degradation behaviors of crystalline Si PV modules with and without a concrete slab was executed via a thermal cycling stress test. The impact of the concrete slab on the performance degradation of PV modules was evaluated. Electroluminescence (EL) results showed that the defect due to thermal cycling (TC) stress was reduced in the PV module with a concrete slab. The power loss due to the thermal cycling was reduced by approximately 1% using a concrete slab for 200 cycles. The *R_sh_* value was reduced to approximately 91% and 71% after thermal cycling of 200 cycles for reference PV modules, respectively. The value of *I_0_* was increased to approximately 3.1 and 2.9 times the initial value for the PV modules without and with concrete, respectively.

## 1. Introduction

Over the years, the photovoltaic (PV) community has put great efforts into reducing the cost per kWh of PV power, which is mainly dependent on the manufacturing costs of the PV system, its efficiency, and its lifetime. In this regard, extensive research work is going into the study of the reliability and durability of PV modules with a focus on their thermomechanical and corrosive degradation under different operating conditions [1,2,3]. In particular, the temperature fluctuation in the PV module heated by solar radiation causes thermal stress at the interfaces between layers, leading to a significant module failure with a degradation rate of up to 0.8% per year [4]. It was reported that the variation in the coefficient of thermal expansion of constituent materials bonded together to form the module induces the thermomechanical stress [5]. Moreover, highly corrosive wet atmospheres, such as marine environments, lead to the corrosion of some metallic parts and the deterioration of the properties of the protective coatings and plastics by the assimilation of salts, causing permanent damages that could impair their functioning [6]. To reduce the testing time under simulated working conditions, specific artificial aging tests were established by the International Electrotechnical Commission (IEC) 61215 and are a key point for efficient and fast development of PV modules optimized for the use in specific climatic conditions [7,8]. The PV module testing procedures under combined climatic and environmental stresses (temperature, thermal cycles, humidity, mechanical load, salt mist, etc.) allow shortening the test time by using simulated test conditions, which are more severe than the actual field operating conditions. Various standard test procedures have been used to evaluate the reliability and identify possible manufacturing defects of the PV modules when operating under standard conditions. Among these tests, thermal cycling (TC) is widely used to profile the progression of degradation, allowing the investigation of the PV module’s reliability of construction, manufacturing processes, and expected field performance. 

Recently, solar-powered roads that incorporate embedded crystalline silicon (c-Si) modules have attracted great interest since they can be used for transportation and power generation. A few examples are finally being rolled out around the world to replace asphalt roads with solar panels [9,10]. However, since the power output of PV modules is affected by various environmental factors, it is necessary to assess their durability/reliability as a function of different aging conditions. Although numerous studies have been conducted [11,12,13], the temperature and corrosion-induced degradation remain important issues, and further work is required to elucidate the response of the PV modules to these aging factors.

In this work, a comprehensive study on the failure and degradation behaviors of c-Si PV modules fixed over a concrete slab is performed via a TC stress test, and a relationship between the device performance and TC duration is established. Combined analysis techniques are employed to examine the degradation mechanisms in the PV modules subjected to the TC stress test. Current–voltage (*I*-*V*) measurements are performed to determine the PV cell parameters (shunt resistance *R_sh_*, series resistance *R_s_*, diode ideality factor *n*, and reverse saturation current *I_0_*) under the illuminated condition concerning the observed degradation of the PV module performance. An electroluminescence (EL) imaging technique is also used for the detection of defects such as microcracks and poor electrical contacts and the analysis of degradation processes that occur during the test. 

## 2. Theoretical

Illuminated *I*-*V* characteristics based on a one-diode model [14,15] of p-n junction solar cells under steady state condition is designated by Equation (1) [16]

(1) $$I=\;I_{ph}-I_{0}\left[e^{\left(\frac{V+IR_{s}}{nV_{T}}\right)}-1\right]-\left(\frac{V+IR_{s}}{R_{sh}}\right)$$

where *I_ph_* is the photogenerated current and *V_T_* (= *kT/q*) is the thermal voltage (*k* = Boltzmann’s constant, *T* = operating temperature, and *q* = elementary electronic charge).

A one-diode exponential model-based analytical method is used to extract the PV cell parameters of the PV modules at a given temperature under illumination conditions [16,17]. The following equations can be derived to extract the values of *R_sh_*, *R_s_*, *n*, and *I_0_*:

(2) $$R_{sh}=\;R_{sc}$$

(3) $$R_{s}=\;R_{oc}-\frac{nV_{T}}{I_{0}}e^{-\frac{V_{oc}}{nV_{T}}}$$

(4) $$n=\;\frac{\left(V_{m}+\;R_{oc}I_{m}-\;V_{oc}\right)}{V_{T}\left\{ln\left(I_{sc}-\;\frac{V_{m}}{R_{sc}}-\;I_{m}\right)-ln\left(I_{sc}-\;\frac{V_{oc}}{R_{sh}}\right)+\left(\frac{I_{m}}{I_{sc}-\;\frac{V_{oc}}{R_{sc}}}\right)\right\}}$$

(5) $$I_{0}=\left(I_{sc}-\;\frac{V_{oc}}{R_{sc}}\right)e^{-\frac{V_{oc}}{nV_{T}}}$$

where *R_sc_* and *R_oc_* are the inverse of the slope (*dI/dV*)^−1^ at short circuit (*V* = 0, *I* = *I_sc_*) and open circuit (*V* = *V_oc_*, *I* = *0*) conditions, respectively. Equations (2)–(5) can be used to extract the values of PV cell parameters using the values of *I_sc_*, *V_oc_*, *R_sc_*, *R_oc_*, *I_m_*, and *V_m_* of single *I-V* characteristics under illumination conditions.

## 3. Experimental Details

The study has been carried out on monocrystalline PV modules (approximately 20 W) with an area of 1540 cm^2^. The rigid modules included 36 cells, with an Al frame (cell area = 31.2 cm^2^). The modules were subjected to a thermal cycling (TC) test to evaluate the ability of the module to withstand thermal mismatch, fatigue, and other stresses caused by temperature fluctuations. The test was conducted using an environmental chamber equipped with an automatic temperature control (−40 to 85 °C). The TC stress was conducted using a chamber (NEC EC−1100, M/s. NET Co. Ltd., Suwon, Korea). First, the temperature of the environmental chamber was decreased from 25 °C to −40 °C in 35 min; then, the temperature was kept constant at −40 °C for 30 min. The temperature was increased from −40 °C to 85 °C in 90 min.

**Figure 1:**
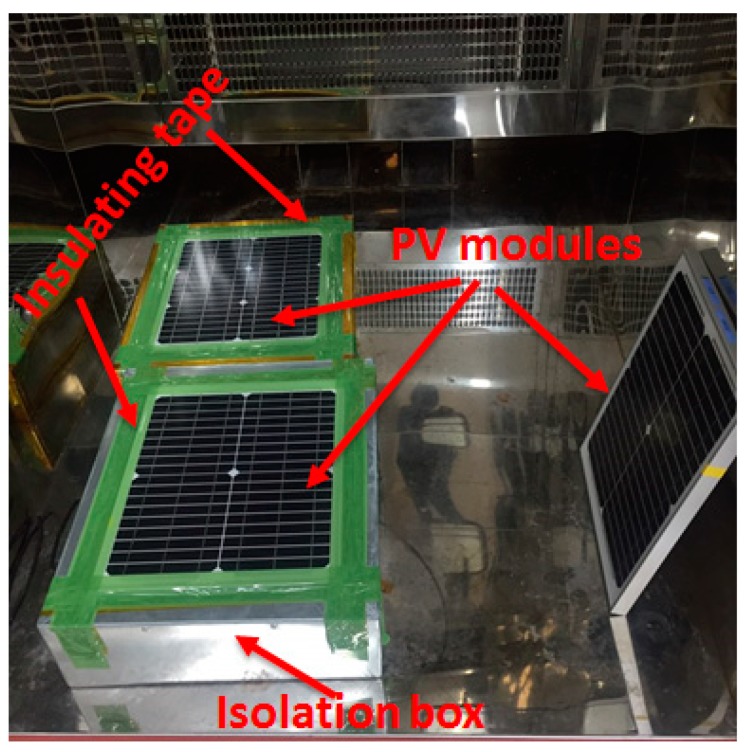
Optical image of photovoltaic (PV) modules inside the thermal cycling chamber.

The PV modules were mounted on a concrete slab to protect the back surface from exposure in an environment [18]. The back surface of the PV modules was isolated from the environment by fixing on the metallic isolation box (filled with concrete), and the gap between the metallic wall and frame of the PV module was covered with insulating tape. The PV module without concrete, in which the backside of the PV module was unprotected, was used as a reference PV module. Figure 1 shows the optical images of PV modules in the thermal cycling chamber. The PV modules were taken out from the environmental chamber after every 20 cycles for *I-V* and electroluminescence (EL) measurements and appearance. 

*I-V* characterization was done after every 20 thermal cycles of each batch. The *I-V* measurements were carried out under a 100 mW/cm^2^ intensity AM 1.5G solar spectrum using a Keithley 2420 source-meter along with a solar simulator (SPI−SUN SIMULATOR 4600SLP, M/s Spire, The Hague, The Netherlands). All measurements were done after stabilizing the temperature of PV modules (at 25 °C). The illumination intensity of the solar simulator was calibrated using a reference Si solar cell (M/s. PV Measurements, Washington, DC, USA). EL scanning was carried out after every 20 cycles using the EL system (TE−2000, M/s. TNETECH, Gyeonggi-do, Korea) equipped with a camera (HR–830SensoCam, M/s. Sensovation, Radolfzell, Germany).

## 4. Results and Discussion

### 4.1. Performance Parameters Analysis

**Figure 2:**
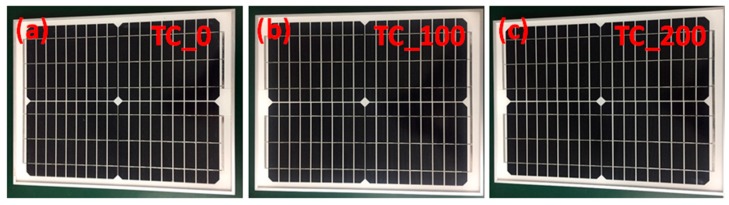
Optical images of reference PV module after thermal cycles of (**a**) 0, (**b**) 100, and (**c**) 200 cycles).

**Figure 3:**
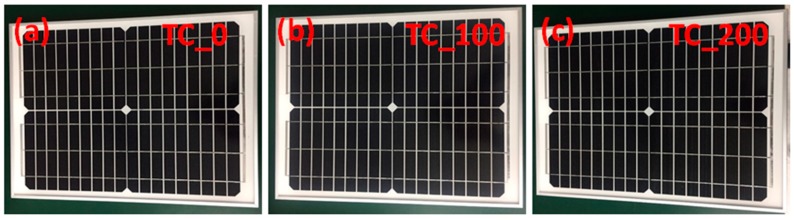
Optical images of the PV module with a concrete slab after thermal cycles of (**a**) 0, (**b**) 100, and (**c**) 200 cycles).

In this study, the c-Si PV modules were fixed on a concrete slab using an isolation box and insulating tape to reduce the heat flow toward the back surface of the PV modules. Thus, two PV modules with a concrete slab were analyzed, while other PV modules without a concrete slab were used as a reference. It is worth noting that the use of the concrete leads to an improvement of the normalized power values compared to that of a reference PV module. *I-V* characteristics were measured periodically (after every 20 cycles) during the TC test (200 cycles) to profile the progression of degradation. The optical images of the reference PV module before and after TC (100 and 200) cycles are shown in Figure 2. However, Figure 3 shows the optical images of the PV module with the concrete slab before and after TC (100 and 200) cycles. For the further investigation of the performance degradation, some of the performance parameters of the PV modules (maximum power *P_m_*, short-circuit current *I_sc_,* open-circuit voltage *V_oc_,* and efficiency *η*) were extracted from the *I*-*V* curves, and their dependency on the thermal cycling was discussed concerning the EL images. 

**Figure 4:**
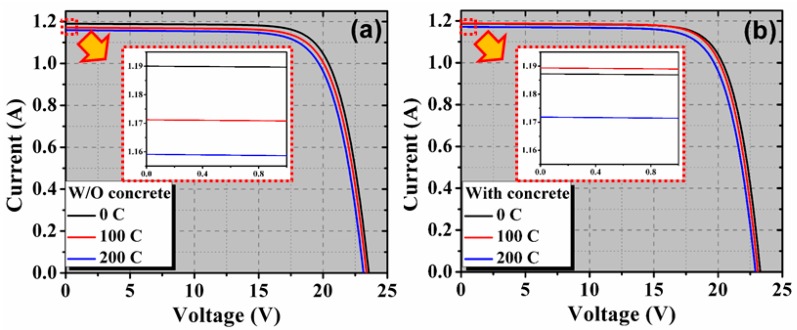
Illuminated *I*-*V* curves after thermal cycling of 0, 100, and 200 cycles for PV module (**a**) without concrete and (**b**) with concrete. The inset shows a magnified part to see the difference in the *I_sc_* values.

**Figure 5:**
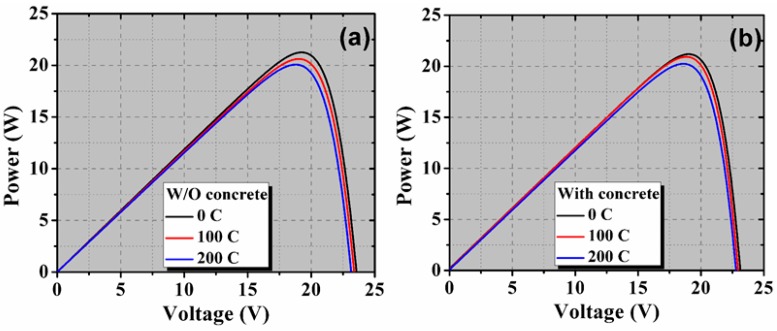
Illuminated *P-V* curves after thermal cycling of 0, 100, and 200 cycles for PV module (**a**) without concrete and (**b**) with concrete.

Figure 4 depicts the measured *I*-*V* characteristics for both the PV modules before and after thermal cycles (100 and 200). These characteristics showed clear signs of series resistance increase, which is mainly attributed to the thermal fatigue undergone by the PV module. The corresponding power-voltage (*P*-*V)* characteristics are shown in Figure 5.

**Figure 6:**
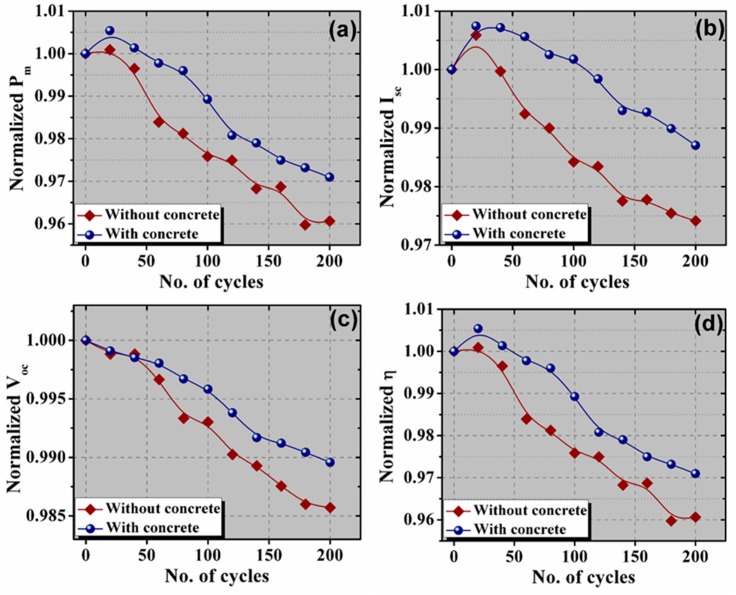
The variation of (**a**) maximum power, (**b**) short circuit current, (**c**) open-circuit voltage, and (**d**) conversion efficiency with thermal cycles.

The values of *P_m_*, *I_sc_*, *V_oc_*, and *η* were determined from the *I*-*V* data, and dependency is illustrated in Figure 6. As can be seen in Figure 6a–c, a reduction in maximum power are observed during the thermal cycle, along with a decrease in *I_sc_* and *V_oc_*. This degradation is evident after 40 cycles with a significant variance in power loss. The *P_m_* value of the PV module with concrete was reduced to 98.9% and 97.1% of its initial value (before TC) after thermal cycling of 100 and 200 cycles, respectively. However, the degradation in the reference PV module was slightly higher than the concrete PV module. The *P_m_* value of the reference PV module is reduced to 97.6% and 96.1% of the initial value, respectively. The *I_sc_* value of the concrete PV module is slightly increased up to 100 cycles, while the reduction in the *I_sc_* of reference PV module started after 40 cycles. This slight improvement in the *I_sc_* might be due to the reduction of the resistive loss caused by proper contact formation. The *I_sc_* values after 200 cycles were reduced by 97.4% and 98.7% for the reference and concrete PV modules, respectively. The *V_oc_* value of both the PV modules started to reduce after 20 cycles. However, the rate of reduction of *V_oc_* value for the PV module with concrete was higher than the reference PV module. After 200 cycles, the obtained *V_oc_* value was reduced by 1.4% and 1.0% for the reference and concrete PV modules, respectively. Moreover, the *V_oc_* and *I_sc_* losses for the concrete PV module were lower compared to those obtained for the reference PV module. The power degradation for the concrete PV module after 200 cycles was approximately 2%, while the maximum power loss measured for the reference module was approximately 3%. These values are below the limit of 5% allowed by the IEC. On the other hand, the *η* value was reduced to approximately 3% and 2% of its initial value for the reference and concrete PV modules, respectively.

**Figure 7:**
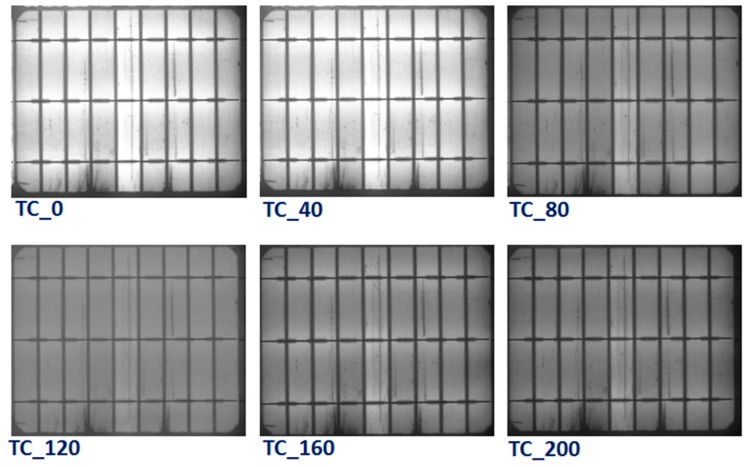
Electroluminescence images of PV modules without concrete after various thermal cycling tests (0, 40, 80, 120, 160, and 200 cycles).

**Figure 8:**
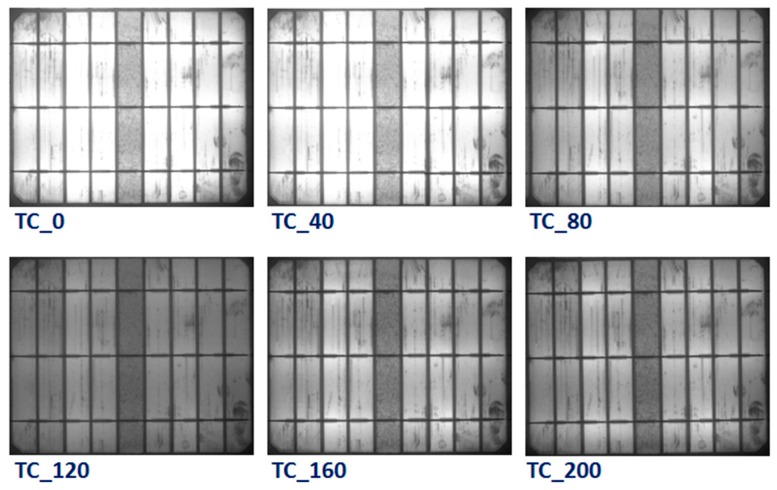
Electroluminescence images of PV modules with concrete after various thermal cycling tests (0, 40, 80, 120, 160, and 200 cycles).

To identify the specific degradation mechanisms and module defects that manifest during the TC test, EL images were taken for all modules. Figure 7 shows the EL images of the reference PV module after different thermal (0, 40, 80, 120, 160, and 200) cycles. There was no obvious change in the EL images of the reference module up to 40 thermal cycles, while the brightness of the EL has diminished in all the areas of the module after 40 cycles. As a result of the extreme temperatures changes during the thermal cycling test, the electrical connections in the PV module are practically stressed. Thus, thermal fatigue-induced defects lead to an increase in the series resistance of the cells, which directly contributes to power degradation. It is well known that the thermal stresses can weaken the electrical connections and soldering in the module and therefore increase the contact resistivity. The serial resistance *R_s_* increases, and consequently, the maximum power of the module decreases. EL images of the PV module with concrete are displayed in Figure 8. The EL results also showed similar behavior with the appearance of dark areas during the TC test. However, the darkness of the EL images in the concrete PV module is lower than the reference module. It can be inferred from these results that a lower number of defects was generated in the PV modules with concrete, which revealed that the impact of TC stress on the PV module containing a concrete slab is smaller than the reference module. This confirms that the impact of the temperature on the PV modules can be reduced through using concrete, which eventually enhanced the performance lifetime of the PV modules.

### 4.2. PV Cell Parameters Analysis

**Figure 9:**
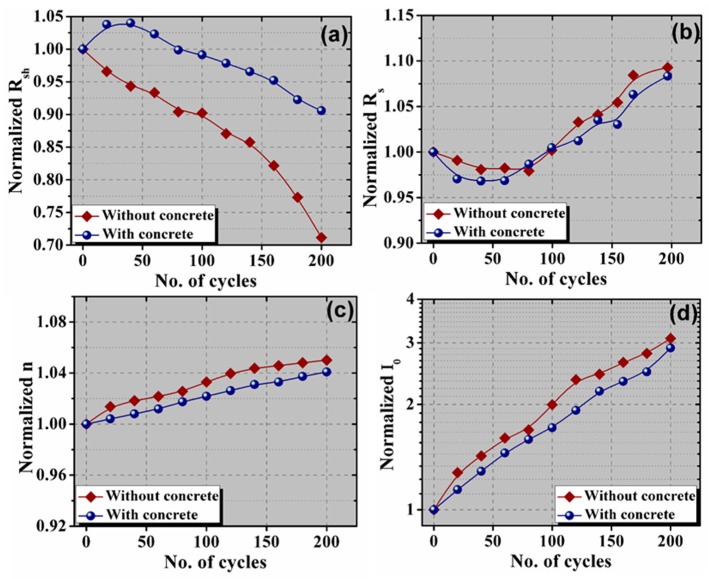
Variation of (**a**) shunt resistance, (**b**) series resistance, (**c**) diode ideality factor, and (**d**) reverse saturation current with thermal cycling.

The performance parameters are directed via PV cell parameters. The origins of the degradation in the performance parameters can be investigated by analyzing the PV cell parameters of the PV cells/modules. The degradation of the performance parameters can also be explained via the PV cell parameters, whose variation is shown in Figure 9. The value of *R_sh_* of the PV module with concrete is initially increased with the increase in the number of thermal cycles. The *R_sh_* value up to 80 cycles is slightly higher than the initial value (before thermal cycling). With a further increase in the thermal cycle, the *R_sh_* value started to decrease. However, the *R_sh_* value of the reference PV module is constantly decreasing with the increase in the thermal cycle. For the reference PV module, both the surface (front and back) are exposed to the environment. However, the back surface of the PV module with a concrete slab is covered, which isolates the back surface from exposure to the environment. Thus, the impact of TC on the PV module with concrete is slightly lower than the reference PV module. Usually, *R_sh_* is due to the parallel current flow through a highly conductive path, which is caused by local defective regions [19]. The defective regions contain a huge number of traps. These traps act as a sink for charge carriers [20,21]. The surface traps can be increased with an increase in the thermal cycle duration [22]. The generation of traps can create inhomogeneity in the vicinity [23]. Thus, the *R_sh_* values are decreased with the thermal cycle, which results in an increase in the leakage current through the p–n junction or from the edge [19]. A low value of *R_sh_* has a miserable impact on *V_oc_*. Therefore, the output power or overall efficiency is reduced. The *R_sh_* value is reduced to approximately 71% and 91% after thermal cycling of 200 cycles. 

In both the PV modules, the value of *R_s_* slightly decreased up to 100 cycles. With the increase in the number of cycles, *R_s_* started to increase. A similar variation in the *R_s_* value is obtained for both the PV modules. After 200 cycles, the *R_s_* value was increased to approximately 108% and 109% for the reference and concrete PV modules, respectively. The *R_s_* of a PV device is the collective resistance of the base material of the PV cell, the contact resistance between PV material and contacts, metallic resistance, etc. Initially, the decrease in the *R_s_* value with the increase in the thermal cycle is mainly due to the reduction of the contact resistance. However, furthermore, the increase in thermal cycling caused the rise in the value of *R_s_* due to the increase in the contact resistance at the metal contact–solder interface. Both the *n* and *I_0_* values are increased with an increase in the number of cycles. However, the variation of the *n* and *I_0_* is lower for the PV modules with concrete. The increment in the value of *n* is 4% and 5% for the reference and concrete PV modules, respectively. Moreover, the value of *I_0_* is increased to approximately 3.1 and 2.9 times the initial value for the reference and concrete PV modules, respectively. *n* and *I_0_* are indicative of the recombination in the PV devices, which rigorously reduced the performance of the PV modules [21,24]. An increase in the n value decreases the value of the curve factor (CF), while *V_oc_* decreases with the increase of *I_0_* [15]. Thus, the overall performance is reduced with the increase in the *n* and *I_0_* values.

## 5. Conclusions

The impact of the concrete slab on the performance degradation of PV modules was evaluated as per IEC standard TC stress testing conditions. EL results showed that the defect due to TC stress was reduced in the PV module with a concrete slab. After 200 thermal cycles, approximately 3% and 2% power loss was obtained for the reference and concrete PV modules, respectively, which revealed that the performance degradation in the concrete PV module was reduced via protecting the backside of the PV modules. The concrete slab can slightly protect the PV module from thermal soaks. Moreover, the *V_oc_* and *I_sc_* losses for the concrete PV module were also lower compared to those obtained for the reference PV module. PV cell parameters were determined using an analytical method based on a single diode model. The obtained PV cell parameters were examined to investigate the loss mechanism. The *R_sh_* value was reduced to approximately 91% and 71% after thermal cycling of 200 cycles for reference PV modules, respectively. However, the *R_s_* value was increased for both the PV modules. Moreover, the value of *n* was slightly increased in both the cases, while the value of *I_0_* was increased to approximately 3.1 and 2.9 times the initial value for the reference and concrete PV modules, respectively.
